# Applications of Alginate-Based Bioinks in 3D Bioprinting

**DOI:** 10.3390/ijms17121976

**Published:** 2016-11-25

**Authors:** Eneko Axpe, Michelle L. Oyen

**Affiliations:** Nanoscience Centre, Department of Engineering, Cambridge University, Cambridge CB3 0FF, UK; mlo29@eng.cam.ac.uk

**Keywords:** alginate, 3D bioprinting, bioink, tissue engineering

## Abstract

Three-dimensional (3D) bioprinting is on the cusp of permitting the direct fabrication of artificial living tissue. Multicellular building blocks (bioinks) are dispensed layer by layer and scaled for the target construct. However, only a few materials are able to fulfill the considerable requirements for suitable bioink formulation, a critical component of efficient 3D bioprinting. Alginate, a naturally occurring polysaccharide, is clearly the most commonly employed material in current bioinks. Here, we discuss the benefits and disadvantages of the use of alginate in 3D bioprinting by summarizing the most recent studies that used alginate for printing vascular tissue, bone and cartilage. In addition, other breakthroughs in the use of alginate in bioprinting are discussed, including strategies to improve its structural and degradation characteristics. In this review, we organize the available literature in order to inspire and accelerate novel alginate-based bioink formulations with enhanced properties for future applications in basic research, drug screening and regenerative medicine.

## 1. Introduction

**Figure 1:**
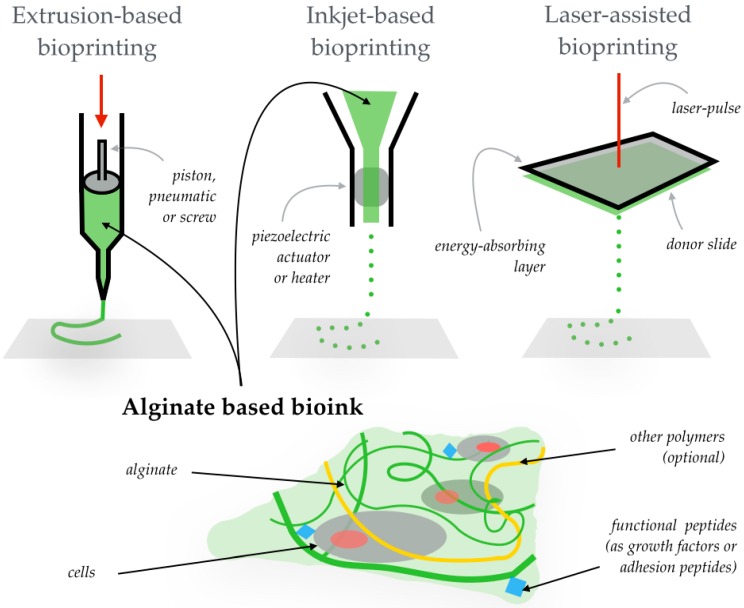
The most widely used bioprinting approaches are shown at the top of the illustration: extrusion-based (performed by a piston, as in the illustration, or by a pneumatic method or a screw), inkjet-based (by a piezoelectric actuator or a heater that creates bubbles) and laser-assisted (with a laser pulse on an energy-absorbing layer that discharges bioink droplets from a donor slide). On the bottom, an illustration shows an alginate-based bioink (composed of the alginate hydrogel, cells, and—optionally—functional peptides to enhance the biological function of the cells, and other polymers forming the hydrogel that tune certain properties (i.e., mechanical or structural) of the bioink and/or the printed three-dimensional (3D) construct).

Three-dimensional (3D) printing aims to integrate living cells in three-dimensional biomaterials. This revolutionary technology permits the automated and reproducible production of 3D functional living tissues by depositing layer-by-layer biocompatible materials (usually containing biochemicals) with a high-precision positioning of cells. This technique permits the fabrication of 3D, scalable and precise geometries that are not offered by other strategies such as two-dimensional (2D) cell cultures or standard 3D cell cultures [1]. There exist three different bioprinting strategies: extrusion, inkjet and laser-assisted (see Figure 1). The uses of these 3D functional living tissues range from basic research [2] (i.e., to study the cell-biomaterial interaction at the nanoscale level—crucial in understanding defects in tissues, organ malfunctioning or nanoparticle-cell interactions [3,4]), drug testing or toxicological studies [5], to real transplantation in animals [6]. Due to the increasing complexity needed for these tissues, 3D bioprinting is facing several challenges in all the production processes. For example, the cell-encapsulated materials are frequently exposed to chemical crosslinkers for extended periods of time during storage prior to printing, which can damage the cells. During the deposition, the mechanical stress caused by the printing itself can result in serious cell damage and loss of cell function by cell extension or shearing [7]. Once the new tissue is printed, the supply of nutrients to cells through the 3D construct is limited, in particular due to the small vascularity of printed materials [8]. In general, the list of requirements for a suitable bioink—or cell-containing dispensable biomaterial—is exhaustive, including printability, biocompatibility, biomimicry and necessary structural/mechanical properties. This is the reason why the vast majority of the manufacturers of commercially available 3D bioprinters—especially those that are extrusion-based—recommend hydrogel bioinks [9].

In this sense, hydrogels are undoubtedly the most extended biomaterials used as cell matrix in bioinks as they can be employed as cell matrix and be tailored to mimic or replace native tissue [10]. The chemical and physical properties of the hydrogels will determine the behavior of the cells. Hydrogels are jelly-like materials in which the liquid component is water. In fact, hydrogels are mostly water by weight, but exhibit no flow in the steady-state due to a 3D cross-linked polymer network within the fluid, which gives them unique properties comparable to those of human tissues. Due to their printability, different biocompatible hydrogels that support cell growth are employed for bioink fabrication: agarose, gelatin, hyaluronic acid, polyethylene glycol (PEG)-diacrylate and alginate, among others.

Alginate is a naturally occurring, non-toxic, biodegradable and non-immunogenic linear polysaccharide composed of guluronic and mannuronic acids [11]. Apart from its high biocompatibility, it is a low-cost marine material—normally obtained from the cell walls of brown algae—that forms hydrogel under mild conditions. For these reasons, numerous materials scientists and bioengineers employ alginate as a component in the design and fabrication of bioinks. The 3D bioprinting of tissues [12] and alginate properties and applications [13] have been recently reviewed separately. Here we review the use of alginate (see Figure 1) in 3D bioprinting.

## 2. The Use of Alginate in Three-Dimensional (3D) Bioprinting

**Figure 2:**
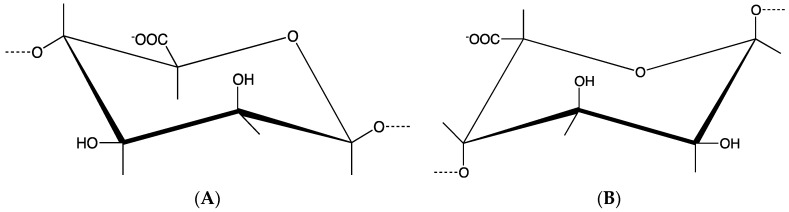
Structural units of the alginate block types: (**A**) β-(1–4)-d-Mannuronic acid; (**B**) α-(1–4)-l-Guluronic acid.

Alginate, also called algin or alginic acid, is a cheap biopolymer normally obtained from calcium, magnesium and sodium alginate salts from the cell walls and intracellular spaces of different brown algae [14]. Alginate is composed of (1–4)-linked β-d-mannuronic (M) and α-l-guluronic acids (G) (see the monomers in Figure 2). Alginate is a polyanionic linear block copolymer made of longer M or G blocks, separated by MG regions. Alginate is a polysaccharide that is negatively charged (it is known that, generally, positively charged materials provoke an inflammatory response). This soluble biopolymer supports cell growth and exhibits high biocompatibility. G blocks increase the gel forming and MG and M blocks increase the flexibility; however, a high amount of M blocks could cause immunogenicity [15]. Water and other molecules can be trapped by capillary forces in an alginate matrix, whereas these molecules are still able to diffuse. This feature makes alginate hydrogels ideal for bioink formulations.

Some 3D bioprinting applications (such as extrusion) require a fast gelation process. By generating ionic interchain bridges, alginate solutions offer fast gelling when mixed with multivalent cations (as Ca^2+^). Though the gelation process in the presence of such cations is not completely understood, it is believed that cations bind both G blocks and M blocks [16]. This way, cells can be easily and quickly encapsulated and interlayer adhesion during the layer-by-layer printing process is avoided [17]. Prior to its use as a bioink, alginate was employed for encapsulating cells in the first therapeutic application of this strategy, in which Langerhan cells microencapsulated in sodium alginate were transplanted into diabetic rats [18,19]. Pore sizes in alginate range between 5 and 200 nm [20], and the largest pores are found in high-G-block-content alginates. This property is important regarding the cell viability of the bioink (due to a limited diffusivity of nutrients) [21].

Each bioprinting methodology requires bioinks with certain rheological properties [9]. Extrusion bioprinting permits the use bioinks of a wide range of viscosities: 30 mPa·s–6 × 10^7^ mPa·s. The cell density within the bioink can be very high, but the shear stress during the extrusion process decreases the cell viability (80%–90%). In inkjet-based bioprinting, the bioinks employed are less viscous (<10 mPa·s) and have lower cell densities (<16 × 10^6^ cells/mL). This method offers cell viabilities of around 90%. The laser-assisted bioprinting requires bioinks with viscosities ranging between 1 and 300 mPa·s and medium cell densities of around 10^8^ cells/mL. The cell viability in this method is very high (>95%). The viscosity of an alginate-based bioink depends on the alginate concentration, the molecular weight of the alginate used (length of the alginate chains), and the cell density and phenotype of the cells. These are the parameters that researchers must take into account in order to tune the viscosity of the alginate-based bioinks. Another important rheological feature of aqueous alginate solutions is the shear-thinning, where the viscosity decreases as the shear rate increases. The viscosity also depends on the temperature at which the printing was performed—the viscosity decreases as the temperature increases. In comparison to other polymers, alginate is reasonably easy to print, as it is easy to handle and extrude while protecting the encapsulated cells. Even though it is non-cell-adhesive by itself [22], in terms of cell encapsulation, alginate is nowadays one of the most utilized materials.

Once the material is printed, the hydrogel should degrade appropriately, permitting the cells to produce their own extra cellular matrix. The alginate creates long-term persistent cell-laden hydrogels, whereas its slow degradation kinetics can be tuned by oxidation (by sodium peroxide, for instance) [23] or by modifying the molecular weight distribution (by gamma rays) of the alginate itself [24]. Alginate lyases catalyze the degradation of the alginate [25]. The degradation of alginate is slow and difficult to control, which is one of the major issues when using this material in 3D bioprinting.

The discharge of the hydrogels during extrusion bioprinting restricts the use to low-weight alginate hydrogels, which, depending on the application, exhibit poor mechanical properties. However, as we will see in further examples, the alginate structural and mechanical properties required for each printed tissue, as well as the biomimicry properties needed in each case, can be tuned by incorporating other biomaterials in the scaffold or by employing different hydrogel fabrication methods. As an example, there already exists a commercially available bioink named CELLINK, which, combining nanocellulose and alginate, presents shear-thinning and fast crosslinking features, making it valuable for soft tissue engineering applications [9]. Moreover, extrusion-based commercially available bioprinters such as Bioscaffolder^®^ from Gesim, or Revolution from Ourobotics, recommend the employment of alginate as a bioink.

In the next section, we will summarizes the most recent advances in 3D bioprinting that have used alginate as (a component of the) bioink.

### 2.1. 3D Bioprinted Vascular Tissues

As isolated cells die in spaces of volumes less than 3 mm^3^ [26], the limited vascularity of the printed materials is a major barrier for 3D organ bioprinting [27]. The creation of blood vessel–like channels capable of transporting, e.g., oxygen and nutrients through the printed material is required in order to fabricate large tissues or organs. To achieve this goal, a coaxial nozzle strategy for nutrient delivery within the printed material was presented by Zhang et al. [28] for the fabrication of vessel-like printable microfluidic channels. In this study, a pressure-assisted bioprinter with a coaxial needle was used to print hollow alginate hydrogel filaments containing cartilage progenitor cells. Similarly, Yu et al. [29] utilized a triaxial nozzle assembly to fabricate biocompatible cartilage-like tissues containing tubular channels. Cartilage progenitor cells were encapsulated in alginate, the main component of the bioink. Gao et al. [30] also obtained printed high-strength sodium alginate hydrogels containing microchannels inside. In a similar fashion, the formation of perfusable vascular constructs was also achieved via a multilayered coaxial nozzle with concentric channel extrusion in one-step 3D bioprinting [31], by blending sodium alginate with gelatin methacryloyl (GelMA) and 4-arm poly(ethylene glycol)-tetra-acrylate (PEGTA). In this work, the crosslinking was made by calcium ions and covalent photocrosslinking of GelMA and PEGTA—used to tune the mechanical and rheological properties. In another study by Christensen et al. [32], vascular-like structures with bifurcations (horizontal and vertical) were printed in sodium alginate and mouse fibroblast–based alginate bioinks. Their inkjet printer was equipped with a calcium chloride solution as a crosslinker and as a supporting material. The solution was used to give a supporting buoyant force for overhang regions in both horizontal and vertical printing, as well as for spanning regions in horizontal printing.

We can conclude from this section that alginate-based bioinks are the most used in coaxial needle–assisted vascular tissue bioprinting, due to the fast ionic crosslinking ability of the alginate. The use of coaxial needles permits tuning the gelation kinetics of alginate-based bioinks with a relatively high precision by adjusting the concentrations of the alginate and the crosslinker.

### 2.2. Bone Printing

Gelatin and alginate, as well as hydroxyapatite, were used to make a novel hydrogel composite for bone printing [33]. A two-step process mixing the thermosensitive properties of gelatin and chemical crosslinking of alginate to achieve fast crosslinking and long-term structural integrity of the 3D-printed constructs with human mesenchymal stem cells was performed during the printing. Hydroxyapatite opens the window of the use of this bioink in bone tissue engineering. Polycaprolatone, which exhibits excellent mechanical properties for bone tissue engineering, was combined with alginate to create 3D osteochondral tissue bioprinting [34]. The combination of both materials reinforced the mechanical properties, a requirement for bone tissue engineering, of the printed 3D construct consisting of osteoblasts and chondrocytes. In a very recent work by Armstrong et al. [35], a bioink was formulated using sodium alginate and a poloxamer as a sacrificial guest, getting bone and cartilage 3D constructs containing porous alginate with enhanced mechanical and rheological properties, even in a microscopic printing definition. Bone-related SaOS-2 cells were 3D bioprinted with gelatin and sodium alginate, and overlayed by agarose and calcium salt of plyphosphate, and they obtained a high cell proliferation and caused an increase in the mineralization of the cells [36]. The same cell phenotype was utilized in a study by Wang et al. [37], in which the effect of bioglass on the growth and mineralization of the SaOS-2 cells was investigated in 3D-printed alginate/gelatin hydrogels. Polyphosphate and biosilica increased the cell proliferation and mineralization. By mixing collagen, polycaprolactone microfibers and nanofibers, and mesenchymal stem cell-laden alginate, Jang et al. [38] fabricated 3D constructs using centrifugal melt-spinning, dip-coating, and bioprinting. The constructs promoted osteogenesis after mastoid obliteration, even in in vivo experiments, accelerating new bone formation. Daly et al. [39] recently presented an interesting strategy. They firstly made cartilage templates using stem cells supported by gamma-irradiated alginate bioink with Arg-Gly-Asp adhesion peptides. Then, the templates were reinforced by printed polycaprolactone, getting a ≈350-fold increase in the compressive modulus which could suppose an advantage in bone tissue engineering.

The mechanical properties of the alginate for bone bioprinting are poor (for instance, the stiffness during elastic deformations of the bone ranges between 15–25 GPa [40], whereas alginate’s is so much lower: 150–550 kPa [41]). We can hereby conclude that the combination of alginate and other polymers such as hydroxyapatite, polycaprolactone, or biosilica, among others, improves the mimicking of the mechanical properties of bone in printed 3D constructs.

### 2.3. Cartilage Printing

Apart from the examples mentioned in the previous section, alginate has been widely employed in cartilage 3D bioprinting. Researchers from the Atala Lab, Winston-Salem, NC, USA [42] designed a combination of electro-spinning and 3D bioprinting, creating layered cartilage with better mechanical properties than the 3D-bioprinted alginate hydrogels. Printed cells produced cartilage extracellular matrix even in vivo. Electro-spinning of polycaprolactone fibers was combined with printing of rabbit elastic chondrocytes encapsulated in a fibrin/collagen gel. In an investigation carried out by Kundu et al. [43], polycaprolactone and alginate encapsulating chondrocyte cells were printed layer by layer to form 3D constructs. Those hydrogels containing transforming growth factor-β (TGFβ) showed a great cartilage-like extra cellular matrix formation. In a work by Markstedt et al. [44], 3D-bioprinted human ears and sheep meniscus were printed using a bioink combining nanofibrillated cellulose and alginate. Combining digital modeling and 3D bioprinting, a meniscus cartilage with a desired pattern was printed in a single-step process [45]. The composite materials were made by combining an alginate/acrylamide solution and an epoxy-based adhesive and extruded a posteriori. This mix was finally cured by UV irradiation. In order to make alginate sulfate printable for cartilage tissue engineering applications, it was combined with nanocellulose by Müller et al. [46], exhibiting good printing properties. Nonetheless, when this bioink was extruded, the chondrocyte cell proliferation was seriously affected when using small-diameter nozzles and valves which limit its application to a low-resolution printing. Further advances in 3D cartilage printing were recently published by Izadifar et al. [47]. The 3D hybrid polycaprolactone and embryonic chick primary cells impregnated with alginate constructs were bioprinted in order to mimic the properties of cartilage.

Alginate is a biostable hydrogel with slow biodegradability and appropriate mechanical properties for cartilage bioprinting, such as PEG, agarose or methylcellulose [48].

### 2.4. Other Advances in 3D Bioprinting

In the previous sections we discussed the problems that the use of alginate presents, and different strategies to face them depending on the application. This information is summarized in Table 1. In the present section, further applications of alginate in 3D bioprinting will be explored.

**Figure 3:**
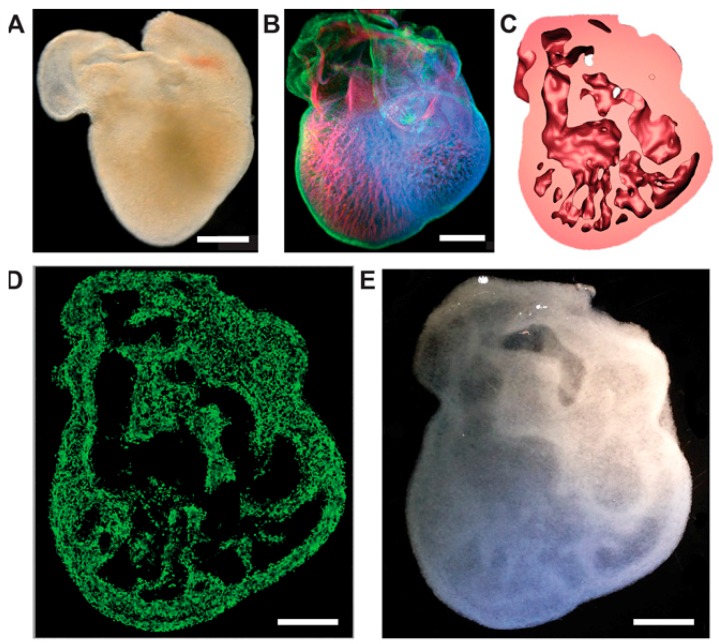
Method to bioprint a trabeculated embryonic heart using alginate-based bioinks. (**A**) Optical microscopy image of an embryonic chick heart; (**B**) a confocal microscopy 3D image of an embryonic chick heart stained for fibronectin (green), nuclei (blue), and F-actin (red); (**C**) a cross-section of the 3D model of the heart based on the confocal imaging data; (**D**) a cross-section of the 3D-printed heart in fluorescent alginate (green); (**E**) optical microscopy image of the bioprinted trabeculated embryonic heart. Figure modified from [55]. Scale bars, 1 mm (**A** and **B**) and 1 cm (**D** and **E**).

Back in 2009, in one of the earliest bioprinting applications, alginate was employed for bioprinting endothelial cells in 3D [50]. In 2010, a direct 3D cell inkjet printer was developed, printing multiple cells with alginate and fibrin hydrogels [51]. In these experiments, alginate showed better mechanical properties but worse cell interaction properties (in terms of cell adhesion, proliferation and differentiation) for tissue growth in comparison to fibrin hydrogel. One year later, sodium alginate hydrogel was used for large tissue fabrication due its quick gelation properties by employing a multinozzle bioprinting system [52]. Regarding other pioneering applications, alginate was employed to constructing the first artificial 3D neural tissue [53]. Specifically, the authors utilized a mix of alginate, carboxymethyl-chitosan, and agarose as a bioink which, once printed, is rapidly crosslinked to form a porous 3D scaffold encapsulating stem cells for in situ expansion and differentiation. This group printed human neural stem cells that were differentiated in situ to functional neurons, forming synaptic contacts that established networks. In the first bioprinting of human-induced pluripotent stem cells and human embryonic stem cells, alginate was also present [54]. The cell response to the valve-based printing and post-printing differentiation into hepatocyte-like cells was investigated. Another breakthrough of the use of alginate-based bioinks was recently published [55]. Complex anatomical structures were made by embedding a printed hydrogel within another hydrogel support. Using models from 3D optical, computed tomography, and magnetic resonance imaging data, femurs, coronary arteries, human brains and trabeculated embryonic hearts (see Figure 3) were bioprinted.

With respect to basic research, in a work by Ning et al. [56], the flow behavior of alginate solutions containing living cells was studied. Rheological properties of alginate–Schwann cell, alginate–fibroblast cell, and alginate–skeletal muscle cell suspensions during shearing in the printing process were analyzed, confirming that the flow behavior effects on cells are critical for their viability and proliferation: in addition to temperature and the concentration of the biomaterial, the cell density affects the flow behavior of cell suspensions too. Three-dimensional bioprinting also offers the possibility of creating realistic tissue models for investigating in vitro 3D biology. Zhao et al. [57] 3D bioprinted a cervical tumor model, with HeLa cells and gelatin/alginate/fibrinogen hydrogel. Comparisons of 3D and 2D results revealed important differences in the biological response of the HeLa cells. By bioprinting alginate and *Escherichica coli* cells, Rodriguez-Revora et al. [49] fabricated a high-throughput drug-screening platform which valuated biochemical reactions in a picoliter-scale volume at a high speed rate and in a cheap way.

One of the challenges of using alginate-based bioinks to bridge the bench-to-bedside translation gap consists of enhancing the biological functions of the bioprinted material. To face this problem, growth factors were incorporated into alginate-bioprinted constructs in an interesting work [58]. Sustained release of bone morphogenetic protein 2 (BMP-2) from the scaffold affected the osteogenicity of the printed tissues. BMP-2 loaded on gelatin microparticles exhibited better release properties in comparison with the direct inclusion of BMP-2 in alginate or bulk gelatin. Another issue of the native alginate is its limited degradation. In a work by Jia et al. [21], the use of oxidized alginates with controlled degradation in 3D bioprinting was investigated. Oxidized alginate solutions with varied biodegradability were printed with human adipose-derived stem cells with high definition. These bioinks were capable of holding a homogeneous cell suspension and modulating proliferation and spreading of the stem cells, but were very limited in terms of diffusion properties. A work by Wu et al. [59] presented a useful method to solve the problem of the slow degradation of alginate hydrogels by incubating the tissues with medium containing sodium citrate. The degradation time of the alginate was tuned by the amount of sodium citrate added. To enhance the printability of alginate, Chung et al. [60] combined gelatin and alginate, enhancing the 3D printability and print resolution of the pre-crosslinked alginate alone, obtaining defined structures with consistent pore diameters which highlighted a higher viscosity and storage modulus while maintaining similar mechanical properties and cell growth.

## 3. Conclusions

Alginate is a low-cost biomaterial which in the form of hydrogel has demonstrated good printability and excellent biocompatibility. It is widely employed in vascular, cartilage and bone tissue printing. However, alginate shows minimal cellular adhesion and slow degradation properties, which in some applications derives in poor cell proliferation and differentiation. Several growth factors (e.g., TGFβ) have been combined to increase the cell proliferation. In order to enhance its cellular adhesion, the addition of Arg-Gly-Asp adhesion peptides to alginate bioink exhibits great results. Furthermore, the uses of oxidized alginate and/or sodium citrate seem to be promising strategies to accelerate the slow degradation of the alginate in regenerative medicine applications. With regard to the employment of alginate in cartilage printing, its combination with electro-spinning has been used in successful works, as well as mixing the alginate with other biopolymers as polycaprolactone or nanocellulose. With reference to the bioprinting of vascularized tissues, the employment of coaxial (or triaxial) nozzle assemblies for printing alginate-based bioinks highlights excellent results. Regarding the mechanical requirements needed for bone tissue engineering, notable improvements have been made by combining alginate with other biomaterials such as gelatin, hydroxyapatite, polycaprolactone, polyphosphate or biosilica. We hope this review will help other researchers improve alginate-based bioinks by employing previous strategies summarized here, or to inspire new bioink formulations for future 3D bioprinting studies.

## Figures and Tables

**Table 1:** Problems and given solutions of using alginate as a bioink in different three-dimensional (3D) bioprinting applications.

3D Bioprinting Application	Problem (of the Use of Alginate)	Solution	Reference
General	Immunogenicity (low cell grow support)	Use a low amount of d-mannuronic acid	[15]
General	Fast gelation needed	Use multivalent cations ^1^	[16]
General	Slow degradation kinetics	Tune the weight percent	[24]
General	Slow degradation kinetics	Oxidation	[23,49]
Vascular tissue	Lack of channels transporting oxygen and nutrients to cells	Use coaxial printing nozzles	[28,29,31]
Bone	Poor mechanical properties	Combination with hydroxypatite	[33]
Bone	Poor mechanical properties	Combination with polycaprolactone	[34]
Bone	Poor adhesion properties	Addition of adhesion peptides (Arg-Gly-Asp)	[39]
Cartilage	Need of biomimetic ECM ^2^	Combination with polycaprolactone 3D constructs	[42,47]
Cartilage	Need of biomimetic ECM ^2^	Combination with nanofibrillated cellulose	[44]
Cartilage	Need of biomimetic ECM ^2^	Combination with acrylamide	[45]
Cartilage	Low printability of alginate sulfate	Combination with nanocellulose	[46]
Cartilage	Low ECM ^2^ formation	Combination with polycaprolactone and growth factors (TGFβ)	[43]

^1^ As the widely used Ca^2+^; ^2^ ECM: extracellular matrix.
